# High-intensity focused ultrasound in the treatment of breast tumours

**DOI:** 10.3332/ecancer.2018.794

**Published:** 2018-01-10

**Authors:** Mirjam C L Peek, Feng Wu

**Affiliations:** 1 Research Oncology, Division of Cancer Studies, King’s College London, Guy’s Hospital Campus, Great Maze Pond, London SE1 9RT, UK; 2 HIFU Unit, The Churchill Hospital, Oxford University Hospitals, Headington, Oxford OX3 7LJ, UK

**Keywords:** high-intensity focused ultrasound (HIFU), breast cancer

## Abstract

High-intensity focused ultrasound (HIFU) is a minimally invasive technique that has been used for the treatment of both benign and malignant tumours. With HIFU, an ultrasound (US) beam propagates through soft tissue as a high-frequency pressure wave. The US beam is focused at a small target volume, and due to the energy building up at this site, the temperature rises, causing coagulative necrosis and protein denaturation within a few seconds. HIFU is capable of providing a completely non-invasive treatment without causing damage to the directly adjacent tissues. HIFU can be either guided by US or magnetic resonance imaging (MRI). Guided imaging is used to plan the treatment, detect any movement during the treatment and monitor response in real-time. This review describes the history of HIFU, the HIFU technique, available devices and gives an overview of the published literature in the treatment of benign and malignant breast tumours with HIFU.

## Background

Breast cancer is the most common cancer in women in the United Kingdom with a one in eight lifetime risk. In 2014, just over 55,000 women were diagnosed and over 11,400 women died from breast cancer, making it the second most common cause of death from cancer in women [1]. Mammographic screening programmes and improved imaging technologies have led to the detection and diagnosis of breast cancer at an earlier stage [2, 3]. However, it has also increased the number of detected benign lesions such as fibroadenomata (FAD). FAD is the most common benign breast lesion in women and the standard of care is reassurance [4]. Active treatment is reserved for symptomatic patients [4]. The standard of care for breast cancer is surgery in the form of either breast conservation or mastectomy followed by adjuvant therapy [5]. However, an increasing number of patients are not satisfied with their cosmetic result after breast surgery. Lesser invasive treatments like radiofrequency ablation, cryo-ablation and laser ablation have become more popular over the years; these techniques show promising results with an improved cosmetic outcome and a decrease in morbidity. Furthermore, for patients with many co-morbidities, lesser invasive treatments offer the opportunity to treat without a life-threatening procedure [3, 6].

High-intensity focused ultrasound (HIFU) is a non-invasive technique that has been used for the treatment of both benign and malignant tumours. With HIFU, an ultrasound (US) beam propagates through soft tissue as a pressure wave with a high frequency. The US beam is focused at a small target volume resulting in a conversion of energy into heat at this site. This increase in temperature causes coagulative necrosis and protein denaturation within only a few seconds [7, 8]. HIFU is capable of providing a completely non-invasive treatment without causing damage to the direct adjacent tissues [7]. This review explains the history of HIFU, the HIFU technique, available devices and gives an overview of the literature published up to December 2016, about HIFU in the treatment of breast tumours.

## History of HIFU

The first therapeutic use of focused US (FUS) was by Bradley *et al* [9], who designed a specially adapted frame and US transducer and successfully used it on patients to treat psychiatric disorders. The study did not continue due to a lack of imaging and the need for craniotomies. In 1962, William Fry and Russell Myers utilised FUS for the treatment of fifty patients with Parkinson’s disease and although the symptoms disappeared, FUS was not used thereafter, due to the development of L-dopa [10]. The first successful use of FUS in breast cancer and thyroid cancer was in 1964 by Oka *et al* [11]. In the 1970s, US was used with lower intensities for the treatment of tumours after the discovery that cancer cells are more sensitive to heat than normal cells [12, 13]. The aim was to induce hyperthermia in the complete tumour lesion and maintain hyperthermia for approximately an hour. Due to a lack of heating and maintenance in the entire tumour volume, caused by a lack of feedback control of the delivered acoustic power to the tumour, this application of FUS did not manage to set grounds [13]. The first clinical application of HIFU was the use of extracorporeal shockwave lithotripsy as a method for treating kidney stones [13] and due to the development of modern technology and advanced imaging methods, interest in HIFU was revived in the 1990s, realising that it can produce instant cell death to the focused areas of tissue. Currently, HIFU has been used in the treatment of both benign and malignant tumours in the liver, breast kidney, uterine and prostate [14].

## The HIFU technique

**Figure 1.**
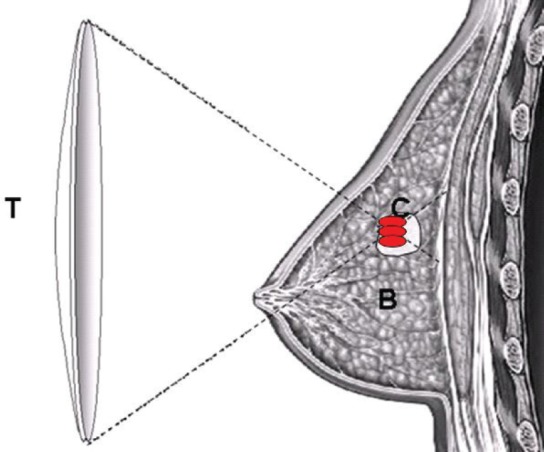
Schematic diagram demonstrating the principle of HIFU ablation for breast tumour. US energy is focused into a small volume in which US energy is converted into heat to induce the required thermal ablation of a targeted breast tumour; (T) – HIFU transducer, (B) – normal breast tissue and (C) – the targeted breast tumour.

In HIFU, a US wave propagates from the transducer through the different tissue layers towards the target site (Figure 1). A part of the energy carried by the sound wave is reflected every time the US wave reaches a tissue boundary, whilst the remaining energy will pass through the tissue layer. The amount of energy that passes through is dependent on the density of the tissue, the speed of sound within tissue layers and the thickness of the tissue layers. It is therefore important to minimize the effect of reflection at the tissue boundaries, as it will otherwise not be feasible to reach the target [14, 15]. When a US wave moves through a soft tissue layer, shearing motion is generated by the induced pressure fluctuations, which results in frictional heating. When a US wave propagates through inhomogeneous media, the wave is scattered in all directions, due to the small regions with different properties within this media, compared to their surroundings, resulting in a loss in acoustic energy [14, 16]. The attenuation coefficient is related to the US frequency and is therefore in most tissues ideal for the use of non-invasive treatment. However, problems arise when the US excitation frequency is increased, which causes both the absorption coefficient and the attenuation coefficient to increase, resulting in a higher heat disposition and a lower penetration depth [14, 16]. As a result, the optimal treatment frequency is dependent on the application; a compromise is needed between the desired penetration depth and the hyperthermia rate [14, 16, 17].

When a US wave propagates linearly through soft tissue, the hyperthermia rate is dependent on the incident US intensity and the local tissue absorption coefficient. Any non-linear mechanism that gives rise to higher frequency components in the US wave will produce enhanced heating due to the frequency dependency of the absorption coefficient previously described. Two mechanisms play an important role in HIFU; non-linear wave propagation and cavitation [14, 18].

In non-linear wave propagation, the US wave becomes gradually shocked resulting in energy loss from the excitation frequency to higher frequencies. The extent of this loss is dependent on the amplitude of the incident wave, the non-linearity of the medium and the travel distance of the US wave. The non-linear effects become more significant in HIFU when there is an increased treatment depth or when a region of high intensity is similar to a region of fatty tissue, which has a higher amount of non-linearity. In HIFU, the heating observed is contributed to significantly by non-linear wave propagation [13, 14].

Small cavities can arise from the thermal effects alone or if the peak rarefaction pressure of a US wave with large amplitude is large enough. When the tissue’s boiling point is reached, large vapour bubbles are formed; the behaviour of these cavities is known as acoustic cavitation, and this process can be divided into two types: stable and inertial cavitation. The type of cavitation is dependent on the size of the bubbles compared to the resonance size, the excitation frequency and the contribution of vapour and gas pressure on the total pressure inside the cavitation. Stable cavitation describes the repeated oscillations of cavities with a size close to or greater than the linear resonance size for the excitation frequency; inertial cavitation describes the explosive growth of cavities with an initial size of about one-third of the resonance size followed by the intense collapse under the effect of the inertia of the surrounding fluid [13, 14, 16]. Cavitation plays a significant double role in US-induced hyperthermia; acoustic energy is being trapped within the cavity due to the strong scattering of the incident wave. This results in enhanced heating due to viscous absorption of the trapped excess energy. In inertial cavitation, the violent collapse of the bubbles as a result of the redistribution of energy leads to more absorption than attenuation, which results in enhanced heat disposition in the surroundings of the cavity [14]. In HIFU, both inertial cavitation and non-linear wave propagation play a significant role in the hyperthermia induced.

## HIFU devices

For the application in breast, two types of image guidance are available, either US or magnetic resonance imaging (MRI). Guided imaging is used to plan treatment, detect movement during treatment and monitor response in real-time.

HIFU guided by US offers real-time visualisation of the treated volume; it can therefore detect any movements made by the patient during the treatment. Furthermore, US can aid in guidance of the energy disposition within the treated area; when applying the first pulse a hyper-echoic cross (hyper echoic [white] area in the shape of a cross) will become visible during the application once the right energy level has been delivered [8, 19]. Furthermore, after application of the pulse a hyper-echoic mark should be visible once the correct energy level is reached. This mark is a representation of the level of coagulative necrosis in real-time during treatment [14]. Other potential benefits of US-guided HIFU are lower cost, increased mobility of the device and wider availability of US [20]. Currently, there are two US-guided devices on the market. The Echopulse device (Theraclion, Malakoff, France) has received a specific CE-mark for the treatment of breast and thyroid nodules. The device includes a cooling and coupling component to cool the skin, in order to prevent any heat-related side effects of the skin such as hyperpigmentation or a skin burn. Cooling does not affect the efficacy of the treatment and can be used for all applications with tumours relatively close to the skin. The device uses a 7.5–12 MHz diagnostic transducer and a 3.0 MHz therapeutic imaging transducer. The transducer ablates a volume of 0.9 × 0.2 × 0.2 cm. A visualisation and treatment unit is connected to the Echopulse and is used for target tissue imaging, power delivery, temperature measurements of the cooling liquid and pressure within the membrane covering the transducer. The skin is cooled during the treatment and cooling liquid is regulated at the membrane and flows from the device towards the probe. Thus far, the Echopulse device has only been used for the treatment of benign breast lesions and not breast cancer.

The second US-guided HIFU device is the Model-JC HIFU system (Haifu Technology, Chongqing, China). This device is CE-marked for the application in solid tumours in soft tissues, including uterine fibroids and adenomyosis, liver tumours, kidney tumours, breast tumours, bone tumours and pancreas tumours. This device uses a 3.5 MHz diagnostic transducer and a 1.6 MHz therapeutic imaging transducer [7, 19, 21]. In breast disease, this device has only been used in the treatment of breast cancer.

Guidance with MRI has the advantage of excellent anatomical resolution, high sensitivity for the detection of lesions and temperature mapping. The high sensitivity of MRI-guided HIFU allows for very precise treatment planning and an accurate evaluation of the efficacy of the treatment at the end of the procedure [20]. However, temperature mapping is complicated due to the high amount of fat and lack of reliability of water proton phase shift-based measurements within fat [22]. Other disadvantages are the high cost, the need for a radiologist and the reduced mobility of the device.

MRI-guided HIFU has been CE-marked for use in uterine fibroids, neurological lesions, adenomyosis and pain palliation of bone metastases; however, the application in breast is still awaiting CE and FDA marking. Currently, the most commonly used devices are the ExAblate 2000 and 2100 systems designed by InSightec-TxSonics Ltd (Haifa, Israel and Dallas, TX) and the Sonalleve MR HIFU system (Philips, Best, The Netherlands). The ExAblate device has been integrated into a 1.5 T MRI scanner (GE Medical systems, Milwaukee, WI) [23]. The device consists of four elements; the MRI unit, the HIFU table, the control personal computer and the HIFU workstation and console. The HIFU table consists of a normal MRI table which is modified to contain a US transducer which is mounted on a mechanical arm and immersed into a water bath. The HIFU workstation consists of a personal computer which communicates with the MRI console computer and the control personal computer and therefore the operator controls the integrated MRI-guided HIFU procedure from the HIFU workstation [24].

A more recently developed system is the Sonalleve MR HIFU system (Philips, The Netherlands), which combines MRI and HIFU in order to be able to perform ablations up to 1.6 × 4.0 cm in volume. It provides temperature-sensitive images in order to determine the location of critical structures and monitor the heat produced. Temperature mapping information is used straight away in order to adjust the treatment; this feedback can help in correcting for local variations in the tissue which can result in inhomogeneous absorption, attenuation and, therefore, overheating. It uses a double membrane with cooled water in order to cool the skin during treatment and measures cumulative heating during the whole treatment. This information is looped back in order to determine the ideal cooling times between pulses reducing the complication rate. Prior to treatment, the patient is placed prone on the treatment table and the target breast is placed into the centre of a ring-shaped MRI surface coil [24]. Pre-treatment MRIs are acquired followed by HIFU treatment. After every sonication the target volume is verified using a temperature-sensitive phase map MRI. After completion of the treatment, another set of MRIs are performed [23].

## US-guided HIFU studies

The first US-guided HIFU studies were performed using patients with breast cancer; however, more recently US-guided HIFU has been used in three trials with breast FAD (Table 1a).

A review by Cavallo Marincola *et al* [20] presented the result of using this device in ten patients with breast FAD. At three months’ follow-up, a 50% reduction in the maximum diameter was seen on US. No adverse events were reported. Kovatcheva *et al* [25] recently reported a study including 42 patients with 51 FAD. A reduction of 59.2 ± 18.2% was seen on US after six months and 72.5 ± 16.7% after one year of follow-up. Skin burns were reported in three patients and hyperpigmentation in one patient. Peek *et al* [26] performed an initial study on 20 patients and 20 control patients with breast FAD. Circumferential HIFU treatment was performed by delivering two treatment rings at the circumference of the lesion and deselecting the centre of the FAD. Circumferential ablation was found to achieve a reduction in treatment time of 37.5 ± 20.1% compared to whole lesion ablation. On US, a significant reduction in FAD volume of 43.5 ± 38.8% (p = 0.016) after six months was observed in the HIFU group and a non-significant reduction of 4.6 ± 46.0% (p = 0.530) in the control group, making the reduction between the two groups significantly different (p = 0.002). In addition, pre-treatment pain resolved after six months in six of eight patients (75%).

Four studies of breast cancer were included in the systematic review by Peek *et al* [27], who were evaluating all HIFU studies performed in breast tumours. The first study, by Wu *et al* [19], included 23 patients treated with HIFU followed by resection of the tumours one to two weeks post-treatment. Staining with haematoxylin and eosin (H&E) showed complete ablation in all patients. A skin burn was the only adverse event reported in one patient. In a second study by Wu *et al* [21], 22 patients were treated with HIFU and followed up for a median period of 54.8 months (range 36–72 months). Core needle biopsies taken at two weeks, three, six and 12 months showed no viable tumour cells in all patients. No adverse events were observed post-treatment, but two patients developed local recurrence after 18 and 22 months post-treatment.

Kim *et al* [7] included six patients with breast cancer. Patients were followed up by dynamic MRI two weeks post-treatment; if no enhancement was visible, patients were followed up with MRI. If nodular or irregular thick enhancement was visible at the ablated tumour periphery, a US-guided biopsy and a second HIFU session were recommended. Complete ablation was seen in 66.7% of patients. Reported adverse events were injury to the pectoralis major (n = 6), nipple depression (n = 1) and skin defect (n = 1). The last study was performed by Guan *et al* [28], who performed a randomised controlled trial and included 25 patients who were treated with the JC HAIFU device. This study showed that HIFU caused coagulative ablation of the tumour and a margin of 1.9 ± 0.4 cm of normal tissue. No complications other than fever in three patients were found.

## MRI-guided HIFU studies

MRI-guided HIFU has only been used once in the treatment of benign breast FAD (Table 1b), by Hynynen *et al* [12] in a small study of nine patients and eleven FAD. Complete ablation was seen in 54% of patients. Pectoralis major injury was reported in one patient.

Ten studies performed MRI-guided HIFU in the treatment of breast cancer. These studies were included in the systematic review by Peek *et al* [27] evaluating all HIFU studies performed in breast tumours.

Huber *et al* [29] performed a case study of a 56-year-old patient with a 2.2 cm tumour. Resection took place five days post-treatment and showed complete ablation of the tumour. Gianfelice *et al* [23, 24, 30] performed three studies with 12, 17 and 24 patients, respectively. In the first study [24], resection was performed immediately post-treatment and showed complete ablation in 17% of patients. Two patients developed a skin burn during treatment. In the second study [23], resection was performed 3–21 days post-treatment and histopathological staining with H&E showed complete ablation in 24% of patients. No adverse events occurred. In the third study [30], patients were followed up for an average of 20.2 months (range 12–39 months). After six months, core needle biopsies were performed, which showed no viable tumour in 58.3% of patients. Ten patients underwent a second HIFU treatment after which another five patients were disease free. In total, complete ablation was obtained in 79% of all patients and one patient developed a skin burn during HIFU treatment.

Zippel *et al* [31] performed HIFU in ten patients and excised the tumour seven to ten days post-treatment. Complete ablation was seen on histopathology (staining unknown) in 20% of patients and two patients developed skin burns during treatment. Khiat et al [32] performed another study with 25 patients. Tumours were resected within 3–21 days and complete ablation was visible in 30% of patients. No adverse events were reported.

Furusawa *et al* [33, 34] performed two studies with 28 and 21 patients. In the first study with 28 patients, tumours were excised five to 23 days post-treatment. Complete ablation was seen in 53.5% of patients and one patient developed a skin burn during treatment. In the second study [34], 21 patients were treated and followed up for a median of 14 months (range 3–26 months). Skin burns were observed in two patients and recurrence was seen in one patient.

Most recently, Cavallo Marincola *et al* [20] performed a non-randomised treat and resect study on ten patients with biopsy-proven single-focus invasive ductal carcinoma. Post-HIFU MRI was performed 10–21 days after HIFU treatment and 14 days after MRI resection of the tumour was planned. The average tumour size was 1.2 cm and the average treatment time was 140 minutes (range 80–180 minutes). No adverse events were reported. In 60% of patients, no enhancement was seen on MRI and this was confirmed on histopathology. In 20% of patients, MRI showed residual enhancing tissue and this was confirmed on histopathology. In 20% of patients, no enhancement was seen on MRI but histopathology showed a small focus of viable cells in the centre of the ablative area. After six months’ follow-up, no evidence of recurrence was seen in any patients on imaging. In addition, Merckel *et al* [35] performed a study in ten patients with early stage breast cancer. Tissue necrosis was observed in six out of ten patients and no complications were found apart from white lumps in one patient. The mean treatment time was 46 ± 17 minutes (12–75 minutes).

## Challenges of HIFU

Although these trials have shown great potential for the use of HIFU in the treatment of both FAD and breast cancer, there are a few challenges which require to be solved before HIFU can be used more widely. Although studies demonstrated high percentages of complete radiological ablation, complete pathological ablation is not consistently obtained in all patients and therefore this technique should be further developed in order to achieve this. The lack of complete histopathological ablation in all patients can be affected by multiple factors. Immobilisation is very important in order to make sure that the treatment is delivered at the correct location, movements due to breathing or pain felt by the patient or due to discomfort caused by the treatment bed are common and should be avoided or reduced to a minimum during treatment. General anaesthesia is not ideal as this brings along additional complications, requires an anaesthetist and prolongs the hospital stay of the patient. However, local anaesthesia was found to be unable to remove all pain caused by the treatment, resulting in movement of the patient [26]. Conscious sedation, a pectoralis major block or a combination of local anaesthesia techniques might be more effective in order to reduce the pain and make the patients more comfortable and relaxed during the treatment. In addition, a well-designed immobilisation covering the complete breast is recommended in order to reduce movements caused by breathing or the heart. Real-time imaging is very important in order to visualise the tumour, follow the progress of the treatment and be able to amend the treatment where necessary. If the quality of the imaging is not high enough the lesion will disappear from the screen and pathological complete ablation will be difficult to obtain as the treatment cannot be followed or corrected when necessary. These difficulties need to be solved before HIFU can be widely used in practice.

Several other ablative techniques are available, such as radiofrequency ablation, laser ablation and microwave ablation, all of which use heat and cryo-ablation which uses freezing to ablate benign and malignant breast tumours. Compared to these techniques, HIFU is the only non-invasive technique as all other techniques require the insertion of needles or probes, which carries additional risks such as skin burns and scarring. Two systematic reviews and meta-analyses were performed, evaluating minimally invasive techniques. [6, 36] These found that HIFU had the highest percentage of technical success, but that in terms of technique efficacy or percentage of complete ablation, techniques as radiofrequency ablation, cryo-ablation and laser ablation are better.

Compared to breast conserving surgery, HIFU does not need general anaesthesia or tissue loss, therefore potentially reducing the risk of procedure-related complications and improving cosmetic outcome.

## Conclusions

Over the years, HIFU has been developed as a non-invasive ablative technique in the treatment of both benign and malignant breast tumours. Tumours can be treated under US or MRI guidance and both modalities show promising results. HIFU should also be further developed in large prospectively conducted clinical trials to validate the efficacy of HIFU compared to surgery.

## Conflicts of interest

The authors declare that they have no conflicts of interest.

## Figures and Tables

**Table 1. (a):** US-guided trials.

Study	n	FAD /BC	Size (cm)*	Age (years)*	Device	CA	Resection	Complications	Time (min)*
**Wu (2003)**	23	BC	3.1 ± 0.8 (2.0–4.7)	46.5 ± 1.7	JC HAIFU	100% (H&E)	1–2 wk	Skin burn (1)	78 (45–150)^1^
**Wu (2005)**	22	BC	3.4 (2.0–4.8)	48.6 (36–68)	JC HAIFU	100% (H&E)	Follow-up 54.8 M (36–72)^1^	Recurrence (2)	132 (60–180)^1^
**Kim (2010)**	6	BC	2.56 (1.2–3.7)	62.1 (46–68)	JC HAIFU	67%	Delayed excision and follow-up 2–30 M	Pectoralis major injury (6), nipple depression (1), skin defect (1)	174.4 (80–285)
**Cavallo-Marincola (2010)**	10	FAD	-	26 (18–34)^1^	JC HAIFU	50% reduction after in maximum diameter after three months	-	Swelling, hardness of area	57.2 (40–100)^1^
**Kovatcheva (2015)**	42 (51)	FAD	3.9 ml (0.3–19.7)^1^	32 (16–52)^1^	Echopulse	Reduction of 33.2 ± 19.1% at two months, 59.2 ± 18.2% at six months and 72.5 ± 16.7% at 12 months	Follow-up 12 M	Skin burn (3), hyper-pigmentation (1)	118 (60–255)^1^
**Guan (2016)**	25	BC	(2.1–4.8)	48 (22–63)	JC HAIFU	-	1–2 wk	Fever (3)	66 (40–132)^1^
**Peek (2016)**	20	FAD	7.3 ± 10.1 cm^3^	30.3 ± 7.5 (18–45)	Echopulse	Reduction of 16.8 ± 19.3% after two weeks, 30.9 ± 52.7% after three months and 43.5 ± 38.8% after six months	Follow-up 6 M	Ecchymosis (9), erythema (6), hypo-pigmentation (1), dimpling (1), numbness (1), skin burn (1), hyper-pigmentation (6)	34.6

**Table 1. (b):** MRI-guided trials.

Study	n	FAD /BC	Size (cm)*	Age (years)*	Device	CA	Resection	Complications	Time (min)*
**Huber (2001)**	1	BC	2.2 × 2.0 × 1.4	56	Unknown	100%	5 D	-	90
**Gianfelice (2003)**	12	BC	2.8 cm^3^ (0.1–8.8)	60 ± 9.6 (45–74)	ExAblate 2000	17% (H&E)	Delayed (unknown time)	Skin burn (2)	80 (35–133)
**Gianfelice (2003)**	17	BC	1.5 cm^3^ (0.1–8.8)	61.2 ± 8.9 (48–76)	ExAblate 2000	24% (H&E)	3–21 D	-	-
**Gianfelice (2003)**	24	BC	1.5 (0.6–2.5)	74.2 (53–92)	ExAblate 2000	79%	Follow-up 20.2 M (12-39)	Skin burn (1)	-
**Zippel (2005)**	10	BC	2.2	56 (45–72)	ExAblate 2000	20%	7–10 D	Skin burn (1)	Max 240
**Khiat (2006)**	25 (26)	BC	3.3 cm^3^ (0.1–11.2)	61.3 ± 11 (45–87)	ExAblate 2000	31%	3–21 D	-	-
**Furusawa (2006)**	28	BC	1.3 (0.5–2.5)	56.9 (41–79)	ExAblate 2000	53.5% (H&E)	5–23 D	Skin burn (1)	140 (76–231)
**Furusawa (2007)**	21	BC	1.5 (0.5–5.0)^1^	54 (34–72)^1^	ExAblate 2000	-	Follow-up 14 M (3–26)^1^	Skin burn (2), recurrence (1)	-
**Cavallo-Marincola (2013)**	10	BC	1.2	-	ExAblate 2000	60%	24–35 D	-	140 (80–180)
**Merckel (2015)**	10	BC	2.0 ± 0.6	54.8 ± 12.5	Sonalleve	-	5.0 ± 2.2 D	White lumps (1)	46 ± 17 (12–75)

* Values are mean ± SD (range), unless indicated otherwise by^1^,

FAD – fibroadenomata, BC – breast cancer, d – days, wk – weeks, m – months

## References

[1] (2014). Breast Cancer.

[2] Fornage BD, Hwang RF (2014). Current status of imaging-guided percutaneous ablation of breast cancer. AJR Am J Roentgenol.

[3] Roubidoux MA, Yang W, Stafford RJ (2014). Image-guided ablation in breast cancer treatment. Tech Vasc Interv Radiol.

[4] Greenberg R, Skornick Y, Kaplan O (1998). Management of breast fibroadenomas. J Gen Intern Med.

[5] Fisher B, Anderson S, Bryant J (2002). Twenty-year follow-up of a randomized trial comparing total mastectomy, lumpectomy, and lumpectomy plus irradiation for the treatment of invasive breast cancer. N Engl J Med.

[6] Peek MC, Ahmed M, Napoli A (2016). Minimal invasive ablative techniques in the treatment of breast cancer: a systematic review. Int J Hyperthermia.

[7] Kim SH, Jung SE, Kim HL (2010). The potential role of dynamic MRI in assessing the effectiveness of high-intensity focused ultrasound ablation of breast cancer. Int J Hyperthermia.

[8] Schmitz AC, Gianfelice D, Daniel BL (2008). Image-guided focused ultrasound ablation of breast cancer: current status, challenges, and future directions. Eur Radiol.

[9] Bradley WG (2009). MR-guided focused ultrasound: a potentially disruptive technology. J Am Coll Radiol.

[10] Fry WJ, Fry FJ (1960). Fundamental neurological research and human neurosurgery using intense ultrasound. IRE Trans Med Electron.

[11] Oka M (1958). Clinical use of ultrasonics and related biological research in Japan. Am J Phys Med.

[12] Hynynen K, Lulu BA (1990). Hyperthermia in cancer treatment. Invest Radiol.

[13] Dubinsky TJ, Cuevas C, Dighe MK (2008). High-intensity focused ultrasound: current potential and oncologic applications. Am J Roentgenol.

[14] Haar GT, Coussios C (2007). High intensity focused ultrasound: physical principles and devices. Int J Hyperthermia.

[15] White PJ, Clement GT, Hynynen K (2006). Local frequency dependence in transcranial ultrasound transmission. Phys Med Biol.

[16] Thanou M, Gedroyc W (2013). MRI-guided focused ultrasound as a new method of drug delivery. J Drug Deliv.

[17] Hill CR (1994). Optimum acoustic frequency for focused ultrasound surgery. Ultrasound Med Biol.

[18] Zderic V, Keshavarzi A, Andrew MA (2004). Attenuation of porcine tissues in vivo after high-intensity ultrasound treatment. Ultrasound Med Biol.

[19] Wu F, Wang ZB, Cao YD (2003). A randomised clinical trial of high-intensity focused ultrasound ablation for the treatment of patients with localised breast cancer. Br J Cancer.

[20] Cavallo Marincola B, Pediconi F, Anzidei M (2015). High-intensity focused ultrasound in breast pathology: non-invasive treatment of benign and malignant lesions. Expert Rev Med Devices.

[21] Wu F, Wang ZB, Zhu H (2005). Extracorporeal high intensity focused ultrasound treatment for patients with breast cancer. Breast Cancer Res Treat.

[22] Kuroda K, Oshio K, Mulkern RV (1998). Optimization of chemical shift selective suppression of fat. Magn Reson Med.

[23] Gianfelice D, Khiat A, Amara M (2003). MR imaging-guided focused ultrasound surgery of breast cancer: correlation of dynamic contrast-enhanced MRI with histopathologic findings. Breast Cancer Res Treat.

[24] Gianfelice D, Khiat A, Amara M (2003). MR imaging-guided focused US ablation of breast cancer: histopathologic assessment of effectiveness – initial experience. Radiology.

[25] Kovatcheva R, Guglielmina JN, Abehsera M (2015). Ultrasound-guided high-intensity focused ultrasound treatment of breast fibroadenoma-a multicenter experience. J Ther Ultrasound.

[26] Peek MC, Ahmed M, Scudder J (2016). High intensity focused ultrasound in the treatment of breast fibroadenomata: results of the HIFU-F trial. Int J Hyperthermia.

[27] Peek MCL, Ahmed M, Napoli A (2015). Systematic review of high-intensity focused ultrasound ablation in the treatment of breast cancer. Br J Surg.

[28] Guan L, Xu G (2016). Damage effect of high-intensity focused ultrasound on breast cancer tissues and their vascularities. World J Surg Oncol.

[29] Huber PE, Jenne JW, Rastert R (2001). A new noninvasive approach in breast cancer therapy using magnetic resonance imaging-guided focused ultrasound surgery. Cancer Res.

[30] Gianfelice D, Khiat A, Boulanger Y (2003). Feasibility of magnetic resonance imaging-guided focused ultrasound surgery as an adjunct to tamoxifen therapy in high-risk surgical patients with breast carcinoma. J Vasc Interv Radiol.

[31] Zippel DB, Papa MZ (2005). The use of MR imaging guided focused ultrasound in breast cancer patients; a preliminary phase one study and review. Breast Cancer.

[32] Khiat A, Gianfelice D, Amara M (2006). Influence of post-treatment delay on the evaluation of the response to focused ultrasound surgery of breast cancer by dynamic contrast enhanced MRI. Br J Radiol.

[33] Furusawa H, Namba K, Thomsen S (2006). Magnetic resonance-guided focused ultrasound surgery of breast cancer: reliability and effectiveness. J Am Coll Surg.

[34] Furusawa H, Namba K, Nakahara H (2007). The evolving non-surgical ablation of breast cancer: MR guided focused ultrasound (MRgFUS). Breast Cancer.

[35] Merckel LG, Knuttel FM, Deckers R (2016). First clinical experience with a dedicated MRI-guided high-intensity focused ultrasound system for breast cancer ablation. Eur Radiol.

[36] Mauri G, Sconfienza LM, Pescatori LC (2017). Technical success, technique efficacy and complications of minimally-invasive imaging-guided percutaneous ablation procedures of breast cancer: a systematic review and meta-analysis. Eur Radiol.

